# Nrf2 Pathway Regulates Multidrug-Resistance-Associated Protein 1 in Small Cell Lung Cancer

**DOI:** 10.1371/journal.pone.0063404

**Published:** 2013-05-07

**Authors:** Lili Ji, Hui Li, Pan Gao, Guoguo Shang, Donna D. Zhang, Nong Zhang, Tao Jiang

**Affiliations:** 1 Department of Pathology, Shanghai Medical College, Fudan University, Shanghai, China; 2 Department of Pharmacology and Toxicology, University of Arizona, Tucson, Arizona, United States of America; 3 Department of Pathology, Medical School of Nantong University, Nantong, Jiangsu Province, China; Helmholtz Zentrum München/Ludwig-Maximilians-University Munich, Germany

## Abstract

Although multidrug-resistance-associated protein-1 (MRP1) is a major contributor to multi-drug resistance (MDR), the regulatory mechanism of Mrp1 still remains unclear. Nrf2 is a transcription factor that regulates cellular defense response through antioxidant response elements (AREs) in normal tissues. Recently, Nrf2 has emerged as an important contributor to chemo-resistance in tumor tissues. In the present study, the role of Nrf2-ARE pathway on regulation of Mrp1 was investigated. Compared with H69 lung cancer cells, H69AR cells with MDR showed significantly higher Nrf2-ARE pathway activity and expression of Mrp1 as well. When Nrf2 was knocked down in H69AR cells, MRP1's expression decreased accordingly. Moreover, those H69AR cells with reduced Nrf2 level restored sensitivity to chemo-drugs. To explore how Nrf2-ARE pathway regulates Mrp1, the promoter of Mrp1 gene was searched, and two putative AREs—ARE1 and ARE2—were found. Using reporter gene and ChIP assay, both ARE1 and ARE2 showed response to and interaction with Nrf2. In 40 cases of cancer tissues, the expression of Nrf2 and MRP1 was measured by immunohistochemistry (IHC). As the quantitive data of IHC indicated, both Nrf2 and MRP1 showed significantly higher expression in tumor tissue than adjacent non-tumor tissue. And more important, the correlation analysis of the two genes proved that their expression was correlative. Taken together, theses data suggested that Nrf2-ARE pathway is required for the regulatory expression of Mrp1 and implicated Nrf2 as a new therapeutic target for MDR.

## Introduction

Multidrug resistance (MDR) is a major obstacle in the treatment of malignant carcinoma. It is a phenomenon in which cancer cells exhibit reduced sensitivity to a large group of unrelated drugs with different mechanisms of pharmacological activity whether it occurs in primary therapy (intrinsic) or is acquired during or after treatment [1]. Mechanistically, the resistance phenomena may be explained by a number of aspects, which include reduced drug accumulation due to the over-expression of transport proteins, increased detoxification, altered targets and impaired apoptosis pathway [2]. One of the most widely studied aspects of MDR is the reduction of intracellular drug accumulation, in which ATP binding cassette (ABC) transporters expressed in the plasma membrane are notorious mediators of MDR [3].

Multidrug-resistance-associated proteints (MRPs) belong to subfamily C of the ABC transporter superfamily (ABCC). In human, the MRP family is composed of several members (MRP1-MRP9), of which, MRP1 plays a very important role in MDR. As one of the drug transporting ABCC proteins, MRP1 was first cloned highly over-expressed in a doxorubicin-selected multidrug resistant human lung carcinoma cell line H69AR [4]. In tumor cells, the 190 kDa MRP1 can confer resistance to not only doxorubicin, but also many other widely used antineoplastic drug, such as methotrexate (MTX), daunorubicin, vincristine and etoposide [5].

Although MRP1 has emerged as an important contributor to chemoresistance, the molecular mechanism for the induction of MRP1 has not been clarified. Several regulatory elements have already been identified to control the expression and inducibility of MRPs [6], [7], [8]. However, there is substantial evidence that indicates the induction of phase II enzymes and MRPs is similar. [9], [10], [11]. As the induction of phase II enzymes is mainly mediated through antioxidant response elements (ARE or EpREs) [12], it suggests that the most likely candidate for a concerted regulation of expression of MRPs is also ARE[13], which has a common 5′-(G/A)TGACnnnGC(G./A)-3′ motif [14]. Recently, in the mouse Mrp2 [15], [16], Mrp3 and Mrp4 gene [16], ARE-like sequences were identified, suggesting the possible role of ARE motif in the regulatory expression of Mrp1. For instance, both γ-GCS, the well-known anti-oxidant enzyme and MRP1 are all induced by oxidative stress [17]. It demonstrated that expression of MRP1 can be up-regulated by redox-active compounds, quercetin in MCF-7 cells [18]. However, the further reporter gene assay indicated that a candidate ARE motif located in the proximal promoter of the Mrp1 gene seemed to be irrelevant for the induction. Later, a putative ARE/AP-1 binding site at −511 to −477 upstream of the transcriptional initiation site in human Mrp1 gene was identified and functioned as a transcriptional enhancer [19]. However, it still did not mediate induction of a luciferase reporter gene upon β-naphthoflavone treatment, which suggests that there might another ARE(s) exist [19]. Although the study using Nrf2 wild type and knockout mouse embryo fibroblasts confirmed that Nrf2 is required for the constitutive and inducible expression of MRP1 [20], how Nrf2 mediates the transcriptional activation of Mrp1 remains unclear, since the exact ARE(s) was not identified yet.

Nrf2 was identified as the main transcription factor that mediates ARE-driven transcription [12]. It regulates the antioxidant response by introducing the expression of genes bearing an ARE in their regulatory regions, such as NQO1, GCS, and HO-1 [21], [22], [23], It is negatively regulated by Kelch-like ECH-associated protein 1 (Keap 1), a substrate adaptor for the Cul3-dependent E3 ubiquitin ligase complex [24]. Activation of the Nrf2 pathway composes a cellular protective system that promotes cell survival under detrimental environments [25], [26]. However, recent studies have shown that constitutively high level of Nrf2 promotes cancer formation and contributes to chemoresistance [27], [28], [29], [30], which is called dark side of Nrf2 pathway. Down regulation of Nrf2 sensitizes cells to chemotherapeutic agents, whereas up regulation enhances resistance in variety of cancer cells [31], [32], [33], [34]. In further support of a role for Nrf2 in chemoresistance, the expression of Nrf2 in cancer cells is increased during acquisition of drug resistance [35], [36]. Collectively, these results demonstrate that Nrf2 contributes to chemoresistance observed in many types of cancer. However, how Nrf2 plays such a role still remains unknown. For instance, which molecular that is tightly regulated by Nrf2 pathway is responsible for chemoresistance? Is MRP1 one of the candidates?

In this study we investigated the regulative role of Nrf2 on Mrp1 expression not only in cultured cell level, but also in cancer patients' tissue. We demonstrated Mrp1 is one of the Nrf2's downstream genes, as we confirmed two ARE motifs in the promoter region of Mrp1 gene. Moreover, there is a high correlation between Mrp1 and Nrf2 expression in different types of malignant tumors. In a word, as more and more attention was paid to the role of Nrf2 in chemoresistance, our study showed the detailed mechanism of how Nrf2 plays its negative role.

## Materials and Methods

### Ethics statement

Permission to use the tissue sections for research purposes was obtained and approved by the Ethics Committee from Shanghai Medical College, Fudan University, China, and a written consent form was obtained from all patients.

### Reagents

Polyclonal antibodies, including anti-Nrf2, Mrp1, Mrp2, Tubulin, β-actin were all purchased from Santa Cruz Inc. (CA, USA). Tert-Butylhydroquinone (tBHQ), sulforaphane (SFN) and all chemicals including cisplatin, doxorubicin and etoposide were purchase from Sigma Co. (St. Louis, MO, USA). Dual-Luciferase Reporter Assay System was purchased from Promega (Madison, WI, USA). All reagents were analytical grade.

### Cell lines, cell culture and cell viability assay

Small cell lung cancer cell line H69, multidrug-resistant small cell lung cancer cell line H69AR, and MDA-MB-231 cell line were purchased from ATCC (Manassas, VA, USA). H69 and H69AR cells were maintained in ATCC-formulated RPMI-1640 Medium, supplemented with 10% and 20% (v/v) fetal bovine serum respectively. MDA-MB-231 was cultured in ATCC-formulated Leibovitz's L-15 medium with 10% (v/v) fetal bovine serum. All cultures were grown at 37°C in 5% CO2 atmosphere. Cell viability was measured by the 3-(4, 5-dimethylthiazol-2-yl)-2, 5-diphenyl tetrazolium bromide (MTT) assay based on the functional change of mitochondria during cell death.

### Recombinant DNA

A pair of primers was designed containing Xho I and Hind III sites to obtain the 992 bp promoter region, from −817 to +175 with numbering relative to the transcription start site (+1), of the human Mrp1 gene from human genomic DNA by PCR. Another four pairs of primers were designed to clone the two possible ARE elements within the promoter region of Mrp1 gene and their mutants accordingly. The amplified products were purified and digested with restriction endonuclease, and then cloned into pGL3-basic (Promega, Madison, WI). The sequences of primers were listed in the table 1.

**Table 1:** Primers used for MRP1 promoter, ARE1,ARE2 and their mutants.

Gene	Primer nucleotide sequences
MRP1 promoter	Forward: CGGCTCGAGTTATCATGTCTCCAGGCTTCA
	Reverse: CGGAAGCTTGCCGGTGGCGCGGG
ARE1	Forward:CAGTATTCACCTCCTTCTGTGTGACTCAGCTTTGGAGTCAGCGGACCGGGC
	Reverse:TCGAGCCCGGTCCGCTGACTCCAAAGCTGAGTCACACAGAAGGAGGTGAATACTGGTAC
ARE2	Forward:CCCACGCCGAGACGCGCGAGGTGAGCGGGCGCCGGGGCGGGGCGGGGTGC
	Reverse:TCGAGCACCCCGCCCCGCCCCGGCGCCCGCTCACCTCGCGCGTCTCGGCGTGGGGTAC
ARE1 mutant	Forward: TCGAGAGTATTCACCTCCTTCTGTG***G***GACT CA***AT***TTTGGAGTCAGCGGACCGGGA
	Reverse:AGCTTCCCGGTCCGCTGACTCCAAA***AT***TGAGTC*C*CACAGAAGGAGGTGAATACTC
ARE2 mutant	Forward:TCGAGCCACGCCGAGACGCGCGAGG*G*GAGCGG***AT***GCCGGGGCGGGGCGGGGTGA
	Reverse:AGCTTCACCCCGCCCCGCCCCGGC***AT***CCGCTCCCCTCGCGCGTCTCGGCGTGGC

The core ARE1, ARE2 and their mutant sequences contained in primers were shown in shade. The mutated nucleotides in the table were in bold italic.

### siRNA transfection and luciferase reporter gene assay

Nrf2 siRNA and Hiperfect transfection reagent were purchased from Qiagen (Valencia, CA) and transfection of Nrf2 siRNA was performed according to the manufacturer's instructions. For luciferase reporter gene assay, cells were transfected with luciferase reporter plasmids for Mrp1 gene promoter, AREs, mutants and renilla luciferase, along with an expression vector for HA-Nrf2. Firefly and renilla luciferase activities were measured using a dual-luciferase reporter gene assay system (Promega, Madison, WI) and analyzed on a GloMax 20/20 luminometer (Promega, Madison, WI).

### Chromatin immunoprecipitation (ChIP)

ChIP analysis was conducted according to protocols provided by Upstate (A Part of Millipore, MA). Briefly, MDA-MB-231 cells or H69AR cells or H69 cells (approximately 4×10^7^) were cross-linked with formaldehyde, collected in PBS, re-suspended in SDS lysis buffer and sonicated on ice. The lysates were then diluted with ChIP dilution buffer, pre-cleared with protein Agarose, and then incubated with indicated antibodies (4 µg/sample) overnight. The immune complexes were collected with protein A agarose, washed and eluted. DNA-protein cross-links were reversed and DNA was recovered. Relative amounts of DNA in the complex were quantified by real-time PCR using LightCycler 480 DNA SYBR green I kit (Roche). Primers shown in Table 2 were designed according to ARE sequences within the promoter region of Mrp1 gene. The confirmed ARE sequence of NQO1 gene was used as positive control for Nrf2 interaction and promoter sequence of Tubulin gene as negative control. The ChIP assay was repeated 3 times.

**Table 2:** Primers used in qRT-PCR amplification.

Gene	Primer nucleotide sequences
NQO1 ARE	Forward: GCAGTCACAGTGACTCAGC
	Reverse: TGTGCCCTGAGGTGCAA
Tubulin promoter	Forward: GTCGAGCCCTACAACTCTATC
	Reverse: CCGTCAAAGCGCAGAGAA
MRP1 ARE1	Forward: AACAGTATTCACCTCCTTC
	Reverse: TAAAGTCTCCACGTTCATG
MRP1 ARE2	Forward: TGCCCACGCCGAGAC
	Reverse: GCAACGCCGCCTGGT
Nrf2	Forward: ACACGGTCCACAGCTCATC
	Reverse: TGTCAATCAAATCCATGTCCTG
KEAP1	Forward: ATTGGCTGTGTGTGGAGTTGC
	Reverse: CAGGTTGAAGAACTCCTCTTGC
MRP1	Forward: TGTGGGAAAACACATCTTTGA
	Reverse: CTGTGCGTGACCAAGATCC
MRP2	Forward: TGAGCATGCTTCCCATGAT
	Reverse: CTTCTCTAGCCGCTCTGTGG
NQO1	Forward: ATGTATGACAAAGGACCCTTCC
	Reverse: TCCCTTGCAGAGAGTACATGG
HO-1	Forward: AACTTTCAGAAGGGCCAGGT
	Reverse: CTGGGCTCTCCTTGTTGC
GST	Forward: TCCCTCATCTACACCAACTATGAG
	Reverse: GGTCTTGCCTCCCTGGTT
TXN	Forward: TCAAATGCATGCCAACATTC
	Reverse: GGTGGCTTCAAGCTTTTCCT
MYC	Forward: CGCTTCTCTGAAAGGCTCTCCTTG
	Reverse: GAGTCGTAGTCGAGGTCATAGTTC
RAS	Forward: AGGCAAGAGTGCCTTGACGATACA
	Reverse: ACTGGTCCCTCATTGCACTGTACT

### Quantitative real-time PCR (qRT-PCR), immunoblot assay

When cells were harvested, the total RNA was extracted using Trizol solution (Invitrogen, Carlsbad, CA). Equal amounts of RNA (2 µg) were reverse-transcribed using the Transcriptor First Strand cDNA synthesis Kit (Roche, IN, USA). The primers used in the next PCR assay were listed in table 2. The qPCR conditions were as follows: one cycle of initial denaturation (95°C for 30 s), 40 cycles of amplification (denaturation at 95°C for 5 s, annealing at 60°C for 30 s, and extension at 72°C for 30 s), followed by a cooling period (50°C for 5 s). Relative mRNA expression was determined using the Rotor-Gene 3000 which employs a modification of the delta–delta Ct method that adjusts for amplification efficiency between target and housekeeping genes. The PCR data were expressed as relative fold change of the target gene between treated and control groups. The cycle point (Cp) values were analyzed by Student's t-test to determine a P value.

For immunoblot assay, cells were homogenized in lysis butter (0.1 M Tris buffer (pH 7.4), 0.1 mM EDTA) in the presence of 1 mM dithiothreitol (DTT), 1 mM phenylmethylsulfonyl fluoride, and protease inhibitor cocktail (Roche, IN, USA). The equal amount of sample was loaded in each well of a 7.5% gel and subjected to SDS-PAGE. Gels were transferred to nitrocellulose membrane. The membrane was then incubated with primary antibodies for 4°C overnight against Nrf2 (110 KD), MRP1(190 KD), and MRP2(∼170 KD). After washing with TBS-T, the membrane was incubated with secondary antibody against Rabbit and mouse IgG and the signals were visualized using ECL plus western blotting system. All experiments mentioned above were performed in triplicate unless otherwise noted.

### Patients and immunohistochemical analysis

The tissues (n = 40) including gastric cancer (n = 10), breast cancer (n = 10), colon cancer (n = 10) and small cell lung cancer (n = 10) were obtained from the Department of Pathology, Shanghai Huashan Hospital, China, within 2007. All cases were diagnosed by 2 pathologists individually in a double-blind manner. The paraffin sections were stained with hematoxylin and eosin (H&E). For immunohistochemical assay, the deparaffinized sections were conducted antigen retrieval by heating in sodium citrate buffer, Ph 6.0. The primary antibody was used in a dilution of 1∶100 (NQO1 and Nrf2) and 1∶50 (Mrp1) for 1 h at 37°C and 4°C overnight. Anti-Nrf2 polyclonal antibody (ab76026) and anti-Mrp1 monoclonal antibody (ab24102) were purchased from Abcam. Monoclonal antibody against NQO1 (SC-271116) was purchased from Santa Cruz. The stained sections were photographed under a light microscope at 400x magnification, and were analyzed by i-Solution software (IMT i-Solution INC., Vancouver, BC, Canada). Five random fields of vision in each sections were selected and analyzed, and the positive area were calculated, which is showed in percentage (ratio of positive area to the whole visual field).

### Statistical analysis

Results were expressed as means±SD. Statistical tests were performed using SPSS Statistics 19. Unpaired Student's t tests were used to compare the means of two groups. One-way ANOVA was applied to compare the means of three or more groups. The Pearson correlation analysis were performed to compare the expression level of Nrf2, Mrp1 and NQO1 by twos. The experimental differences were determined by two-tailed Student's t-test and p<0.05 was taken as significant difference in all cases.

## Results

### In contrast with H69 cells, H69AR cells has higher Nrf2 pathway activation and Mrp1 expression level

**Figure 1:**
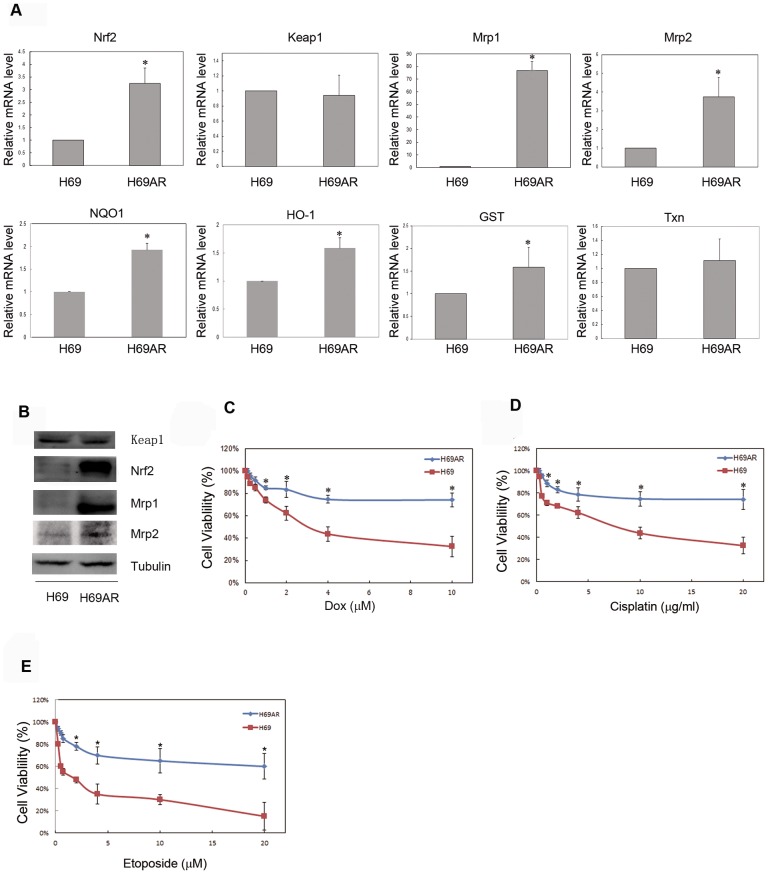
Nrf2 involves in the expression of Mrp1 and MDR in H69AR cells. (A) Relative mRNA level of Nrf2, Keap1, Mrp1, Mrp2, together with NQO1, HO-1, GST and Txn was compared in H69 and H69AR cells by real-time RT-PCR using the ΔΔCt method with GAPDH as an internal control. Results were expressed as mean ± SD from three independent experiments. (B) Protein level of Keap1, Nrf2, Mrp1, Mrp2 was compared in H69 and H69AR cells by Western blotting using Tubulin as internal control. Experiments were performed three times and a representative immunoblot was shown here. (C), (D) and (E) The viability of H69 and H69AR cells with treatment of Dox, Cisplatin and Etoposide in different doses for 24 hours was determined by MTT assay. The experimental differences were determined by two-tailed Student's t-test and p<0.05 was taken as a significant difference in all cases.

On basal level, the activation of Nrf2 pathway was compared between H69 and H69AR cells. The downstream genes of Nrf2, such as NQO1, HO-1 and GST showed increased mRNA expression in H69AR cells, but there was no significant difference in Txn mRNA expression (Figure 1, panel A, * P<0.05 vs. H69 cells). Interestingly, there was a higher Nrf2 mRNA level in H69AR cells than H69 cells, with no difference in Keap1 gene (Figure 1, panel A, * P<0.05 vs. H69 cells). Both Mrp1 and Mrp2 gene showed higher mRNA level in H69AR cells, especially Mrp1 had more than 70 fold in H69AR cells to H69 cells (Figure 1, panel A, * P<0.05 vs. H69 cells). Next the protein level of Nrf2 was measured in the two cell lines. Consistent with the changes in mRNA level, H69AR cells had higher Nrf2 pathway activation than H69 cells (Figure 1, panel B) in protein level. Moreover, Mrp1 and Mrp2 also showed increased expression in H69AR cells (Figure 1, panel B). Interestingly, the major negative regulator of Nrf2, Keap1 did not show any difference between the two cell lines (Figure 1, panel B). Considering the important roles that Mrp1 and Mrp2 play, H69AR cells have multi-chemo drugs resistance due to the high level of Mrp1 and Mrp2. As our MTT data indicated, H69AR cells had more viability rate with chemo drugs treatment, such as Doxorubicin, Cisplatin and Etoposide (Figure 1, panel C, D and E, * P<0.05 vs. H69 cells).

### Knockdown of Nrf2 leads to decreased Mrp1 expression and sensitized H69AR cells to multi-chemo drugs

**Figure 2:**
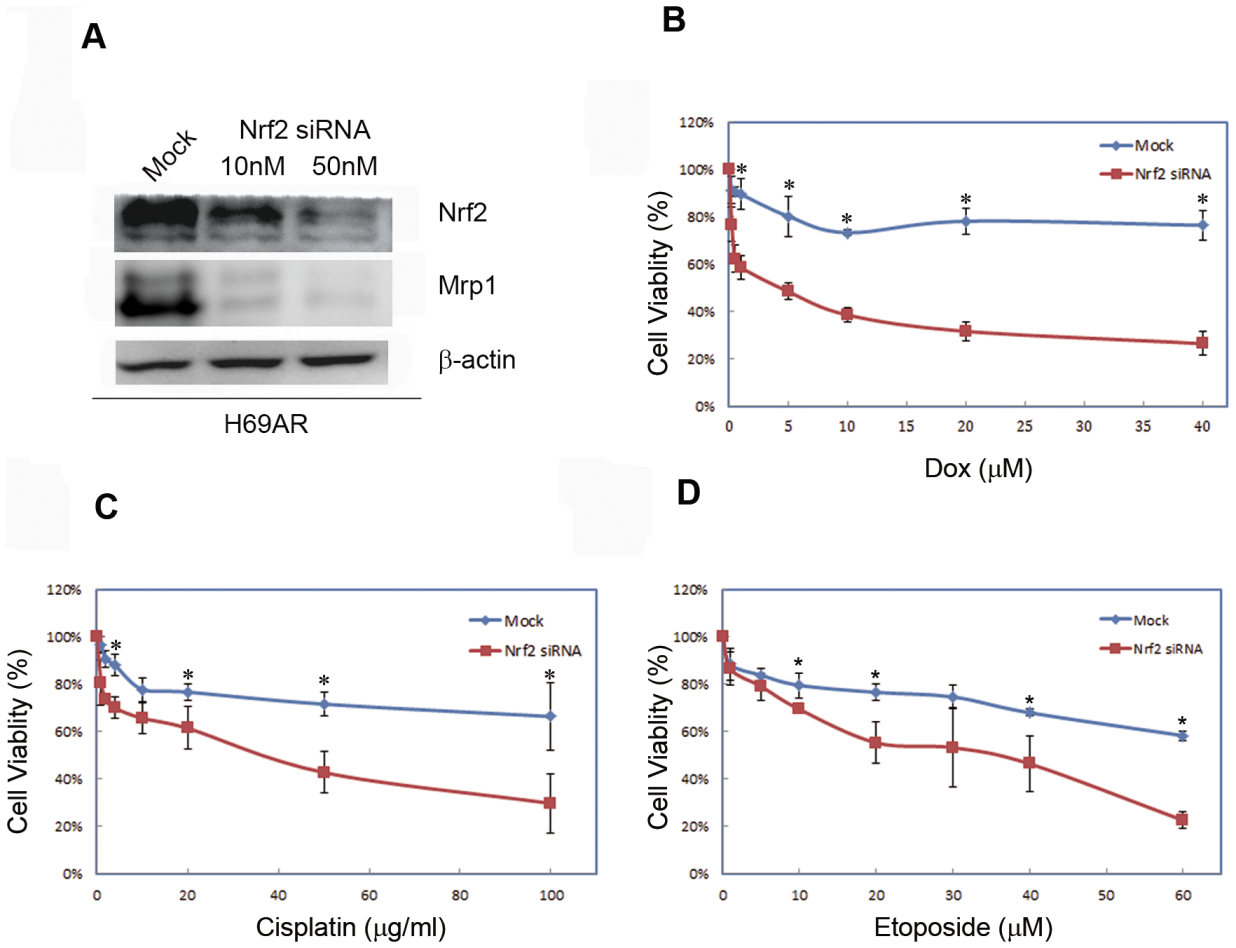
Down regulation of Nrf2 leads to decreased Mrp1 and sensitized H69AR cells to chemotherapeutic drugs. (A) H69AR cells were performed transfection of Nrf2 siRNA (10 nM and 50 nM) for 72 hours. Protein expression of Nrf2 and Mrp1 was determined by western blotting using β-actin as internal control. Experiments were performed three times and a representative immunoblot was shown here. (B), (C) and (D) H69AR cells were subjected to knockdown of Nrf2 with siRNA transfection. Forty-eight hours later, cells were treated with Dox, Cisplatin and Etoposide in different doses for additional 24 hours, and they were then subjected to MTT assay to measure cell viability. The experimental differences were determined by two-tailed Student's t-test and p<0.05 was taken as a significant difference in all cases.

To analyze the regulation of Mrp1 by Nrf2, specific siRNA was used to knock down the Nrf2 expression. As the data showed, the expression of Nrf2 was reduced more than 50% in contrast with the mock group (Figure 2, panel A). More importantly, the protein level of MRP1 was also reduced accordingly (Figure 2, panel A), which means Mrp1 may be regulated by Nrf2 pathway. As H69AR showed multi-chemo drugs resistance, next the resistance was measured again when expression of Nrf2 was knocked down. As expected, H69AR cells restored sensitivity to all the three chemo drugs including Doxorubicin, Cisplatin and Etoposide, when the specific siRNA was used to reduce significantly the expression of Nrf2 (Figure 2, panel B, C and D, * P<0.05 vs. mock group). Collectively, these data indicated the possible role of Nrf2 on regulation of Mrp1 and confirmed the role of Nrf2 in chemo-resistance.

### The 5′-flanking region of the human Mrp1 gene contains two ARE sequences

**Figure 3:**
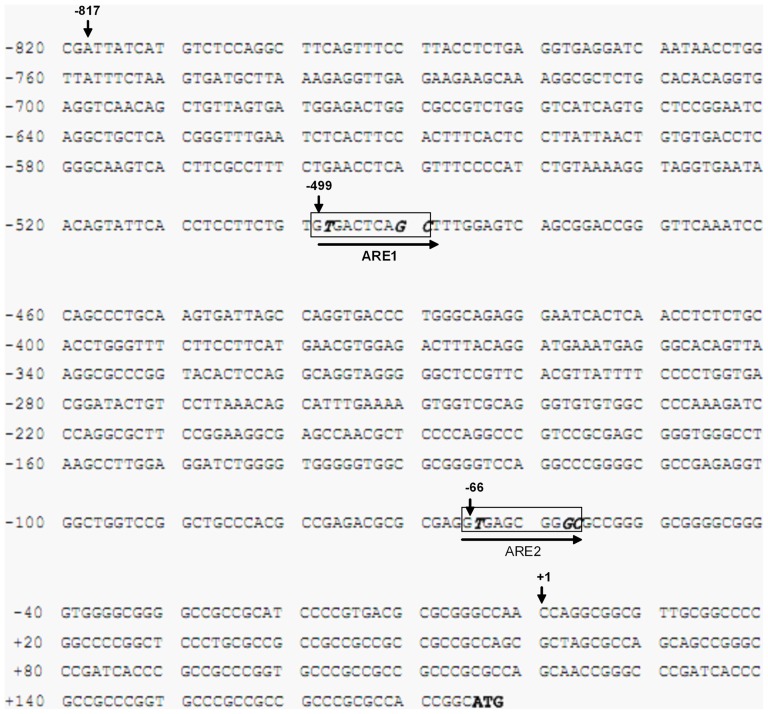
Nucleotide sequence of the 5′-flanking region of the hMrp1 gene containing two potential ARE sites. Nucleotides were numbered relative to the transcription start site (+1). The translation initiation codon (ATG) was in bold. Boxed region indicated ARE1 and ARE2 respectively. The nucleotides to be mutated in this study were in bold italic.

To determine the presence of ARE sequence in the 5′-flanking region of the human Mrp1 gene, a 992-bp long fragment was cloned and sequenced from nucleotides −817 to +175 with numbering relative to the transcription start site (indicated as +1 in Figure 3). The nucleotide sequence of the 5′-flanking region of the human Mrp1 gene was shown in Figure 3. Transcription start site was set to be at 5′-end of the Mrp1 mRNA sequence with the longest 5′-untranslated region, and was localized 175 base pairs upstream of the translation start site (indicated in bold in Figure 3). A number of cis-acting sequences were identified. Interestingly, two ARE-like sequences were found in the isolated regions: one at positions −499 to −490 (ARE-1; gTGActcaGC, with lower-case letters indicating non-conserved bases) and the other at positions −66 to −57 (ARE-2; gTGAgcggGC). The two ARE sequences were identical with the previously reported minimal ARE enhancer sequence (a/g)TGA(C/T/G)nnnGC [37]. The nucleotides to be mutated were highlighted in bold italic in Figure 3.

### Nrf2-mediated induction of Mrp1 promoter activity depends both on ARE1 and ARE2

**Figure 4:**
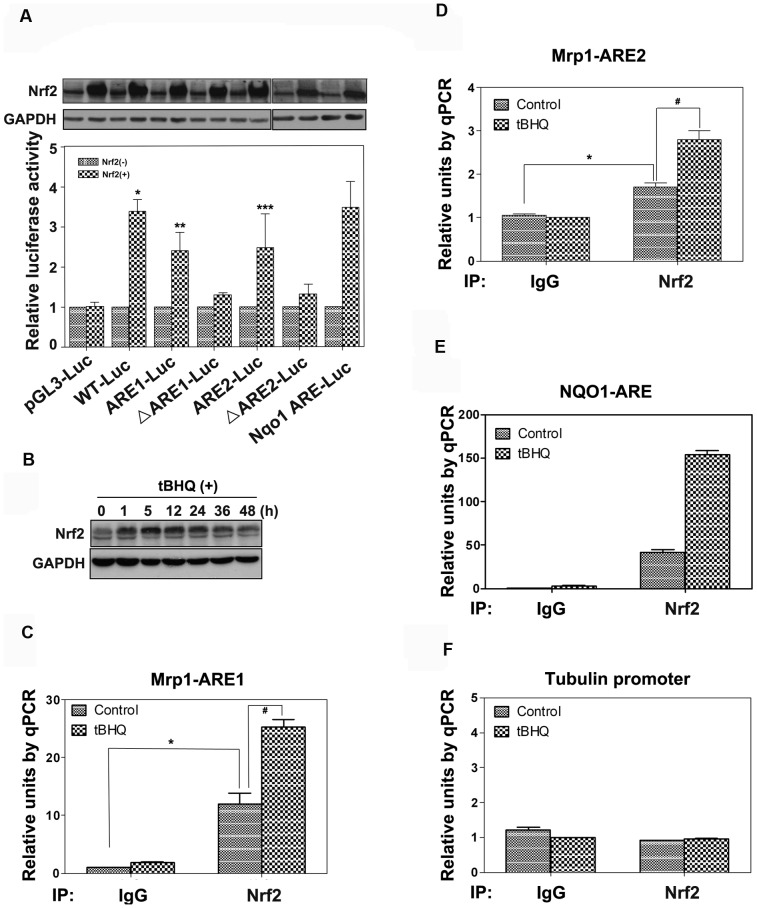
Nrf2-mediated induction of Mrp1 promoter activity depends both on ARE1 and ARE2 in 231 cells. (A) 231 cells were transfected with five luciferase reporter constructs (wt-Luc, ARE1-Luc, ARE2-Luc, ΔARE1-Luc and ΔARE2-Luc), and promoterless pGL3-basic (pGL3-Luc), NQO1-ARE-Luc and pRL-TK were used as negative control, positive control and internal control, respectively. The aforementioned plasmids were co-transfected with or without Nrf2-expression plasmid. After 48 hours, cells were harvested and luciferase activities were measured with a dual-luciferase assay system (lower panel). Data were means ± SD of values from three independent experiments. The equal amount of transfection of Nrf2 was assured by western Blot (upper panel). (B) 231 cells were treated with 40 µM tBHQ for the indicated time point, and then were harvested to be subjected to immune blot to measure Nrf2 protein level. Experiments were performed three times and a representative immunoblot was shown here. (C), (D), (E) and (F) 231 cells were treated or not with 40 µM tBHQ for 24 h and were cross-linked with formaldehyde. Sonicated chromatin was subjected to immunoprecipitation with antibodies against Nrf2 or normal IgG. Precipitated DNA was followed by realtime PCR analysis with primers against Mrp1 ARE1 (panel C), Mrp1 ARE2 (panel D), NQO1 ARE (panel E) and Tubulin promoter (panel F). Experiments were performed three times and a representative of three experiments was shown here.

To investigate the response of the 5′-flanking region of Mrp1 to Nrf2, dual luciferase reporter gene assay was performed. Firstly, the 992-bp fragment was amplified by PCR from human genomic DNA with primers that was listed in Table 1, and then the PCR product was cloned into pGL3-basic vector and named pGL3-wt. Secondly, based on pGL3-wt, the two putative AREs and mutated AREs were amplified and cloned into pGL3-basic vector, which were named pGL3-ARE1, pGL3-ARE2, pGL3-ΔARE1 and pGL3-ΔARE2 (The mutated nucleotides were highlighted in bold italic in Table 1). The reporter assay demonstrated that Nrf2 induced the promoter activity of Mrp1 gene after co-transfection of pGL3-wt with Nrf2 expressing plasmid (Figure 4, panel A, * P<0.05 vs. control). It also indicated that the induction of Mrp1 promoter activity was associated with the two putative AREs, since both of them were up-regulated by overexpression of Nrf2. Moreover, mutation of them ablated the induction accordingly (Figure 4, panel A, ** P<0.05 vs. control, ***P<0.05 vs. control). However, there was no significant difference of induction between the two AREs. In addition, NQO1 ARE-Luc was used as a positive control to assure the validity of the whole assay, and the immunoblot assay was performed to make sure that the overexpression of Nrf2 was quite equivalent (Figure 4, A, upper panel).

**Figure 5:**
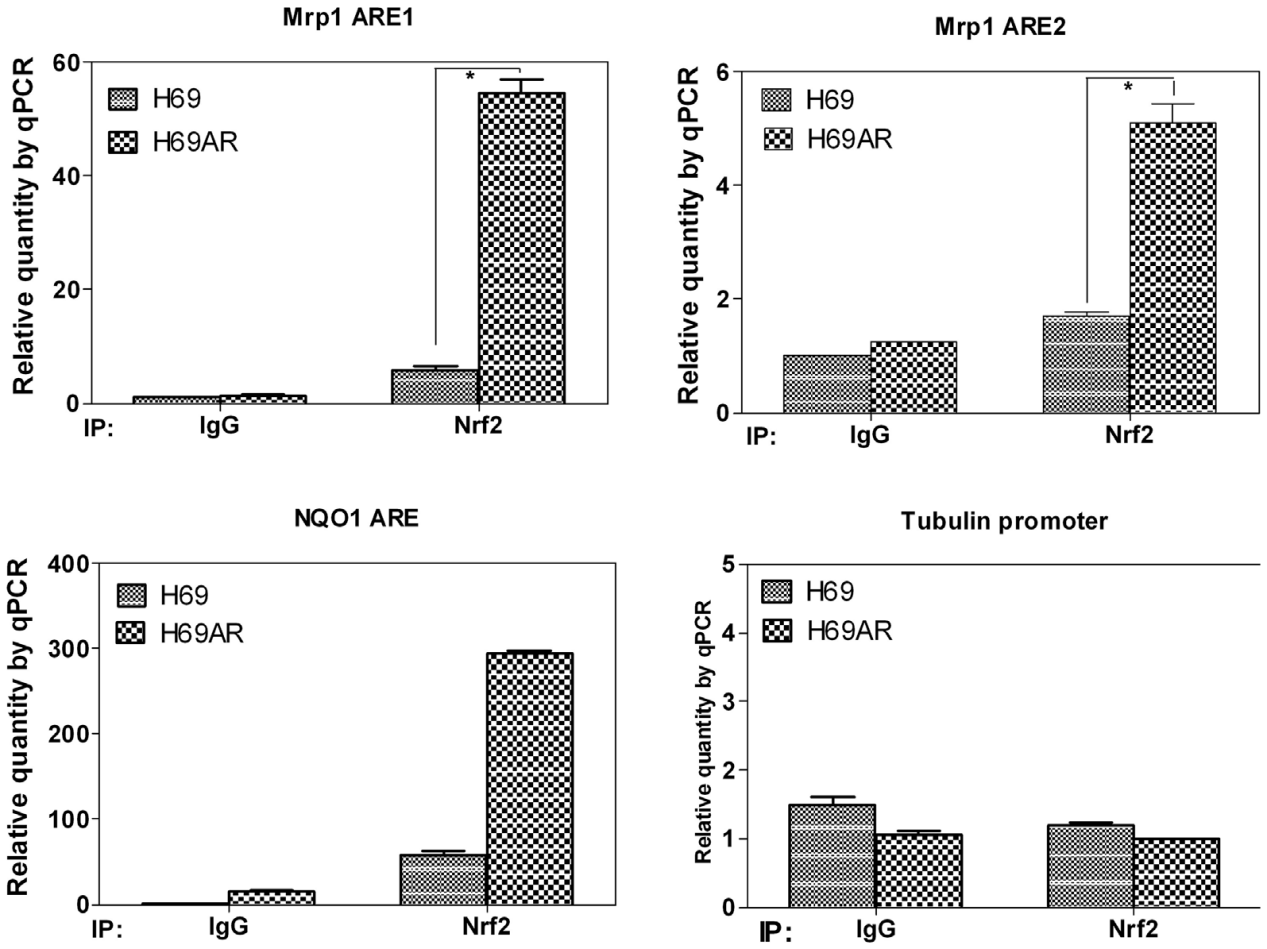
Nrf2-mediated induction of Mrp1 promoter activity depends both on ARE1 and ARE2 in small cell lung cancer cells. H69 and H69AR cells were cross-linked with formaldehyde. Sonicated chromatin was subjected to immunoprecipitation with antibodies against Nrf2 or normal IgG. Precipitated DNA was followed by realtime PCR analysis with primers against Mrp1 ARE1 (panel A), Mrp1 ARE2 (panel B), NQO1 ARE (panel C) and Tubulin promoter (panel D). Experiments were performed three times and a representative of three experiments was shown here.

Next, to confirm the true binding of Nrf2 with the two putative AREs, ChIP assay was performed both in 231 cells and small cell lung cancer cells. According to the immunoblot assay of Nrf2 treated with tBHQ in 231 cells, the well-known Nrf2 pathway inducer, an appropriate treating time of tBHQ, 24 hours was set (Figure 4, panel B). So 231 cells (4×107) were treated with 40 µM tBHQ for 24 hours, and then were harvested to subject to ChIP assay. Compared with the negative control, the binding with ARE1 by Nrf2 was increased significantly (Figure 4, panel C, * P<0.05). Moreover, treatment with tBHQ made the binding increased much more higher (Figure 4, panel C, ^#^ P<0.05). Similarly, ARE2 also showed binding with Nrf2 with or without tBHQ treatment (Figure 4, panel D, * P<0.05, ^#^ P<0.05). However, the binding of Nrf2 with ARE2 was lower than ARE1 in both basal level and tBHQ treatment level. As the expression of Nrf2 was much higher in H69AR cells than H69 cells (Figure 1, panel B), we next performed a comparative CHIP analysis to test whether increased Nrf2 in H69AR cells was bound to ARE1 and ARE2 elements in Mrp1 promoter. Consistent with our expectation, the binding with ARE1 by Nrf2 was increased significantly in H69AR cells compared with H69 cells (Figure 5, panel A, * P<0.05). Similar binding results with ARE2 was also found (Figure 5, panel B, * P<0.05). This may suggest a molecular explanation for increased MRP1 expression in H69AR cells. Moreover, binding of Nrf2 with ARE2 was lower than ARE1 in both H69 and H69AR cells, which was consistent with the results in 231 cells. The positive and negative controls of ChIP assay assure the experiment was believable and the data was solid (Figure 4, panel E and F and Figure 5, panel C and D). Collectively, these data demonstrated that the two putative AREs within Mrp1 gene promoter region were real AREs that could be regulated by Nrf2.

### The expression of Nrf2 and Mrp1 was correlative and higher in malignant tumors than adjacent non-tumor tissue

**Figure 6:**
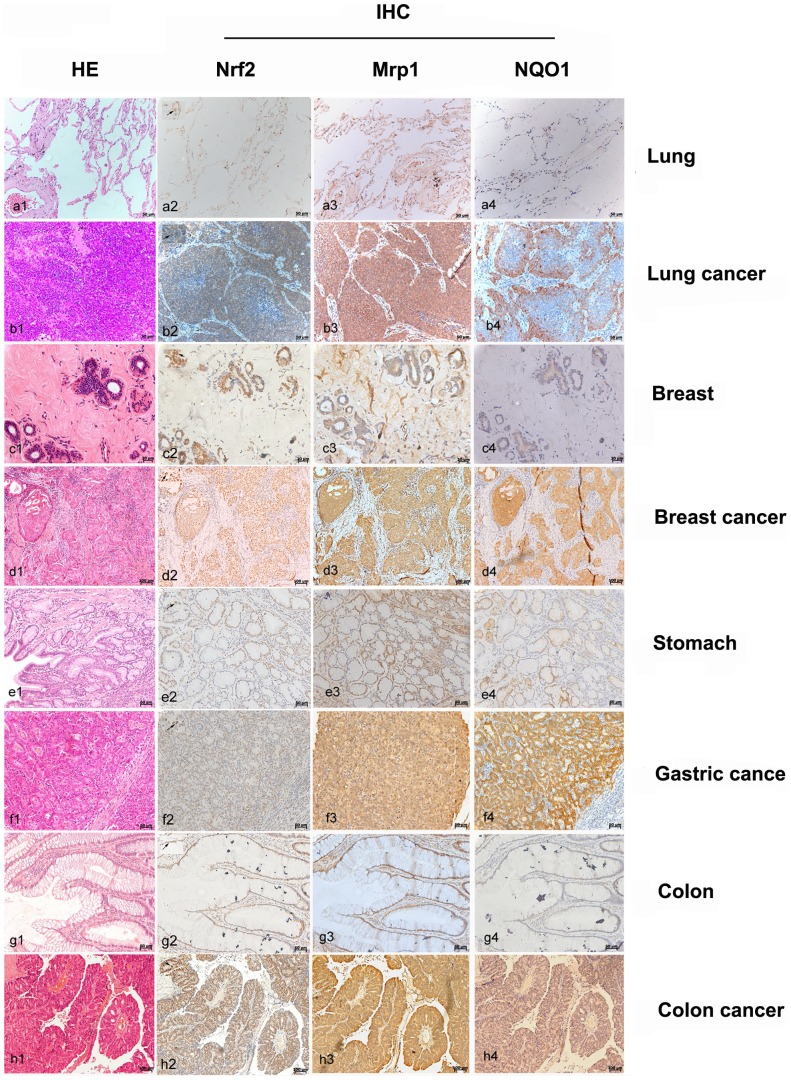
Immunohistochemical staining results for Nrf2, NQO1 and Mrp1 expression in cancer tissuess. The unstained tissue slides from small cell lung cancer, gastric cancer, breast cancer and colon cancer were subject to HE staining (a1 to h1) and IHC staining with antibodies against Nrf2 (a2 to h2), Mrp1 (a3 to h3) and NQO1 (a4 to h4). The bar scales were labeled at the lower right corner of each pictures.

Totally, 40 cases of malignant tumors, including lung cancer, breast cancer, gastric cancer and colon cancer, were selected to measure the *in vivo* expression of Nrf2, Mrp1, and NQO1 as well by IHC. For lung cancer, only the cases that were diagnosed with small cell lung carcinoma (SCLC), where both H69 and H69AR cells originated form, were selected. Compared with the adjacent non-tumor tissue, the lung cancer tissue showed dramatically higher expression of Nrf2, Mrp1 and NQO1 (Figure 6, compare panel b2 to a2, b3 to a3, b4 to a4). The HE staining in the panel of left most revealed the malignant hyperplasia and adjacent non-tumor tissue, which is helpful to localize the positive signal in IHC-stained sections. In adjacent non-tumor tissue, Nrf2 mainly expressed in the nuclei of epithelial cells (Figure 6, panel a2), and the expression of Mrp1 was showed both on cell membrane and in cytoplasm (Figure 6, panel a3). To show the nuclear localization of Nrf2, a selected area within each photo of Nrf2 staining was enlarged and merged into the original photo at the upper left corner (Figure 6, panel a2-h2, black arrow). It was hard to see any obvious NQO1 expression except faint positive signal in scattered cells in adjacent non-tumor tissue (Figure 6, panel a4). In lung cancer tissue, the expression of Nrf2 was limited in cancer cell nodules except the necrotic center cells. Both cytoplasm and nuclei showed Nrf2 expression, which was much stronger than adjacent non-tumor tissue (Figure 6, panel b2). In a similar expressing pattern with Nrf2, Mrp1 also expressed obviously and diffusely in the nodules of lung cancer (Figure 6, panel b3). As one downstream gene of Nrf2, the expressing pattern of NQO was similar with Nrf2 (Figure 6, panel b4). It was hard to see any expression of Nrf2, Mrp1 and NQO1 in interstitial tissue. In breast cancer tissue, the expression of Nrf2, Mrp1 and NQO1 was significantly high (Figure 6, panel d2, d3 and d4), though mild expression of them could be seen in adjacent non-tumor tissue (Figure 6, panel c2, c3 and c4). The three genes in gastric cancer and colon cancer were alike in expressing pattern, namely the expression of them in tumor tissue was much higher than in adjacent non-tumor tissue (Figure 6, panel e1 to h4). Moreover, the IHC staining data indicated the possible correlation between Nrf2 and Mrp1 expression.

**Figure 7:**
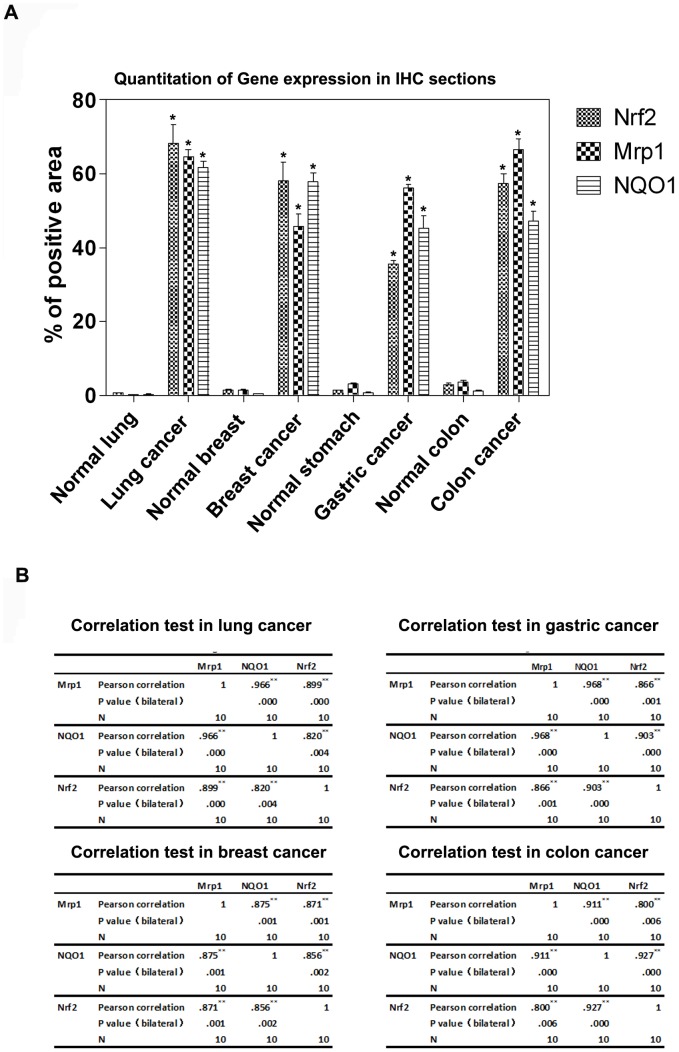
Statistical analysis of IHC data using i-Solution software. Five random visual fields were selected in each section and then were taken pictures of by camera. The pictures were analyzed by i-Solution software, and the positive area and the whole area of each picture were calculated. The protein level of Nrf2, Mrp1 and NQO1 was expressed as ratio of positive area to whole area. (A) The different expressing levels of Nrf2, Mrp1 and NQO1 in the four cancers were compared. (B) The correlation analysis was performed by SPSS between Nrf2, Mrp1 and NQO1 by twos.

Next, the IHC staining sections were analyzed and measured by software, i-Solution to quantitate the expression of Nrf2, Mrp1 and NQO1 in all cases. The expression was presented as the ratio of positive area to whole area. In all the four cancer tissues, Nrf2, Mrp1 and NQO1 showed significantly higher expression in contrast with the adjacent non-tumor tissue (Figure 7, panel A, * P<0.05 vs normal tissue, respectively). To confirm the correlation of their expression by twos, the Pearson correlation test was next performed. In lung tissue, including both tumor tissue and adjacent non-tumor tissue, the Pearson correlation coefficient (r) between Nrf2 and Mrp1 equals to 0.899 (Figure 7, panel B, upper left, ** P<0.001), which means the expression of the two genes was correlative. As one of the downstream genes of Nrf2, the expression of NQO1 is regulated by Nrf2, so the r equals to 0.820 (Figure 7, panel B, upper left, ** P<0.005), which confirmed the regulation of NQO1 by Nrf2. Like the correlation of Nrf2 and Mrp1 in lung tissue, all other 3 tissues showed this significant correlation too, such as stomach tissue (Figure 7, panel B, upper right), breast tissue (Figure 7, panel B, lower left) and colon tissue (Figure 7, panel B, lower right). Therefore, these staining data and statistical analysis demonstrated both Nrf2 and Mrp1 were expressed much higher in tumor tissue than adjacent non-tumor tissue, and more important, the expression of the two genes was correlative.

## Discussion

As one of the 9 MRP members (MRP1-MRP9), MRP1 is a major contributor to chemoresistance which remains the pivotal limitation in the treatment of cancer patients, especially after surgery. The involvement of MRP1 in MDR to oncolytic drugs has been confirmed through several in vitro and in vivo studies [38], [39], [40], [41], [42]. However, the molecular mechanism for the regulation of Mrp1 still remains unclear. Considering the key role of Nrf2-ARE pathway in induction of phase II enzymes [12], it may also regulate expression of Mrp1, since the parallel expression of both phase II enzymes and MRPs was confirmed [9], [10], [11].

Nrf2 bears dual roles in cancer. Although lots of cancer preventive compounds from diet have been proved Nrf2 inducers, it is upregulated in most cancer cells and responsible for acquired chemoresistance [27]. Overexpression of phase II enzymes is not the only culprit thought to be involved in the chemoresistance. The transporters, such as MRPs, may contribute more to chemoresistance, since they are responsible for exporting the drugs out of the cell. The recent emerging reports demonstrated that Nrf2-ARE pathway is responsible not only for phase II enzymes, but also MRPs, for instance, there is AREs found in the promoter region of MRP2 gene [15]. Furthermore, Nrf2 was shown to be necessary for the inducible expression of MRP1 in MEFs [20]. Our study confirmed the regulative role of Nrf2 on expression of MRP1 both in lung cancer cells and cancer tissues, including lung, breast, colon and gastric cancer. Moreover, Nrf2 upreglates the transcription of MRP1 through binding to its 2 defined ARE sequence, which suggests the different sensitivity to chemo drugs between H69 and H69AR cells is due to the Nrf2-ARE-MRP1 level.

Although MDR transporters are generally considered to be cell surface localized, where they are thought to reduce the cellular accumulation of toxins or chemo drugs over time, some reports have also suggested the subcellular localizations for P-glycoprotein (Pgp), MRP1 and breast cancer resistance protein (BCRP)[43], [44], [45]. Moreover, SM. Simon's group demonstrated that members of MDR transporter family, including MRP1, are also expressed in subcellular compartments where they actively sequester drugs away from their cellular targets[46]. Their study showed that intracellular localization and activity for MRP1 and other members of the MDR transporter family may suggest different strategies for chemotherapeutic regimens in a clinical setting. In our data, the expression of MRP1 in different cancer tissues was observed both on cell membrane and in cytoplasm. This phenomenon is consistent with previous reports, and it may suggest the functional complexity of MRP1 in chemoresistance.

In the past decades, uncovering transcriptional regulators of MRP1 has been stimulated by research seeking molecular targets that could modulate the chemoresistant phenotype and thereby constitute an effective therapeutic treatment. ARE is not new for regulation of MRP1 gene, since Kurz's group had identified one in human MRP1, which is we named as ARE1 in our study [19]. In Kurz's study, ARE1 was responsible for the higher transcription of Mrp1 in H69AR cells than H69 cells in basal level, though treatment of β-naphthoflavone (β-NF), one Nrf2 inducer [47], [48], did not show any induction of MRP1. Therefore, either the “ARE” is not real ARE that can bind to Nrf2, or there might exist other ARE(s) in Mrp1 gene promoter region that account for the huge difference in gene expression upon treatment of Nrf2 between H69 and H69AR cells. In our study, we found a new fragment with constitutional ARE sequence and is more close to transcription start site than ARE1. We proved this fragment is a real ARE, and it is responsible for activation of Nrf2 by tBHQ. Although data from Kurz's group indicated no response of ARE1 with treatment of β-NF, ours showed it did have induction after tBHQ treatment. We think the inconsistence may result from different compound and cell types used. They used HepG2 cells, but we used MDA231 cells. The difference in ARE1 and ARE2 was that the latter interacted less with Nrf2 than ARE1, though both of them showed interaction with Nrf2 in ChIP assay and similar inductive folds in reporter assay. In conclusion, both the two AREs within Mrp1 gene promoter region play roles in Mrp1 transcription not only in basal level but also after inducing by Nrf2, though ARE2 responds less than ARE1.

As we confirmed the regulation of Nrf2 on MRP1 gene in the cell, the correlation of them was analyzed in human tissue. It is true that Nrf2 showed significantly higher expressing level in tumor tissue than adjacent non-tumor tissue, which proved the negative role of Nrf2 in cancer again. Moreover, based on the quantitative analysis of IHC staining, the correlation analysis was performed, and the role of Nrf2 in Mrp1 regulation was confirmed again especially in small cell lung cancer, where both H69 and H69AR cell originates from. The correlatively high expression of Nrf2 and its downstream genes, such as Mrp1 and NQO1, endues the malignant tumors with more power to survive when facing stimulus, such as chemo-drugs and radiation. This is the essence of the dual role of Nrf2, and should be concerned when a whole strategy of chemotherapy is set. Actually, more and more groups have been focusing on this negative effect of Nrf2 and trying to explore ways to inhibit Nrf2 in order to acquire better efficacy.

In conclusion, we found that Nrf2-ARE pathway is required for the regulatory expression of Mrp1 in H69AR cells, and the expression of Mrp1 is correlative with Nrf2 in both tumor tissue and adjacent non-tumor tissue. Both Nrf2 and Mrp1 is dramatically higher in H69AR cells than H69 cells, the interesting thing is there is no significant difference on Keap1 level between them. As the negative regulator of Nrf2, Keap1 controls the protein level of Nrf2. This is the well-known molecular mechanism of regulation of Nrf2 and has been always focused on. However, the transcriptional regulation of Nrf2 may also be important. For instance, we found that mRNA level of Nrf2 is quite higher in H69AR than H69 cells. Actually DeNicola GM et al have reported that k-ras induces transcription of Nrf2 to promote ROS detoxification and tumorigenesis [49]. We also proved c-myc regulates Nrf2 transcription in H69AR cells (*unpublished data*). Consequently, the transcriptional regulation of Nrf2 should also be noticed besides the post-translational regulation, and needs to be further investigated.

## References

[1] Fojo AT, Ueda K, Slamon DJ, Poplack DG, Gottesman MM, et al (1987) Expression of a multidrug-resistance gene in human tumors and tissues. Proc Natl Acad Sci U S A 84: 265–269. 2432605 10.1073/pnas.84.1.265 PMC304184

[2] Baird RD, Kaye SB (2003) Drug resistance reversal–are we getting closer? Eur J Cancer 39: 2450–2461. 14602131 10.1016/s0959-8049(03)00619-1

[3] Szakacs G, Paterson JK, Ludwig JA, Booth-Genthe C, Gottesman MM (2006) Targeting multidrug resistance in cancer. Nat Rev Drug Discov 5: 219–234. 16518375 10.1038/nrd1984

[4] Cole SP, Bhardwaj G, Gerlach JH, Mackie JE, Grant CE, et al (1992) Overexpression of a transporter gene in a multidrug-resistant human lung cancer cell line. Science 258: 1650–1654. 1360704 10.1126/science.1360704

[5] Borst P, Evers R, Kool M, Wijnholds J (2000) A family of drug transporters: the multidrug resistance-associated proteins. J Natl Cancer Inst 92: 1295–1302. 10944550 10.1093/jnci/92.16.1295

[6] Tirona RG, Kim RB (2005) Nuclear receptors and drug disposition gene regulation. J Pharm Sci 94: 1169–1186. 15858847 10.1002/jps.20324

[7] Klaassen CD, Slitt AL (2005) Regulation of hepatic transporters by xenobiotic receptors. Curr Drug Metab 6: 309–328. 16101571 10.2174/1389200054633826

[8] Zhou J, Zhang J, Xie W (2005) Xenobiotic nuclear receptor-mediated regulation of UDP-glucuronosyl-transferases. Curr Drug Metab 6: 289–298. 16101569 10.2174/1389200054633853

[9] Kuo MT, Bao J, Furuichi M, Yamane Y, Gomi A, et al (1998) Frequent coexpression of MRP/GS-X pump and gamma-glutamylcysteine synthetase mRNA in drug-resistant cells, untreated tumor cells, and normal mouse tissues. Biochem Pharmacol 55: 605–615. 9515571 10.1016/s0006-2952(97)00494-2

[10] Ishikawa T, Bao JJ, Yamane Y, Akimaru K, Frindrich K, et al (1996) Coordinated induction of MRP/GS-X pump and gamma-glutamylcysteine synthetase by heavy metals in human leukemia cells. J Biol Chem 271: 14981–14988. 8663001 10.1074/jbc.271.25.14981

[11] Theodoulou FL, Clark IM, He XL, Pallett KE, Cole DJ, et al (2003) Co-induction of glutathione-S-transferases and multidrug resistance associated protein by xenobiotics in wheat. Pest Manag Sci 59: 202–214. 12587874 10.1002/ps.576

[12] Itoh K, Chiba T, Takahashi S, Ishii T, Igarashi K, et al (1997) An Nrf2/small Maf heterodimer mediates the induction of phase II detoxifying enzyme genes through antioxidant response elements. Biochem Biophys Res Commun 236: 313–322. 9240432 10.1006/bbrc.1997.6943

[13] Meijerman I, Beijnen JH, Schellens JH (2008) Combined action and regulation of phase II enzymes and multidrug resistance proteins in multidrug resistance in cancer. Cancer Treat Rev 34: 505–520. 18413281 10.1016/j.ctrv.2008.03.002

[14] Rushmore TH, Morton MR, Pickett CB (1991) The antioxidant responsive element. Activation by oxidative stress and identification of the DNA consensus sequence required for functional activity. J Biol Chem 266: 11632–11639. 1646813

[15] Vollrath V, Wielandt AM, Iruretagoyena M, Chianale J (2006) Role of Nrf2 in the regulation of the Mrp2 (ABCC2) gene. Biochem J 395: 599–609. 16426233 10.1042/BJ20051518 PMC1462684

[16] Maher JM, Dieter MZ, Aleksunes LM, Slitt AL, Guo G, et al (2007) Oxidative and electrophilic stress induces multidrug resistance-associated protein transporters via the nuclear factor-E2-related factor-2 transcriptional pathway. Hepatology 46: 1597–1610. 17668877 10.1002/hep.21831

[17] Yamane Y, Furuichi M, Song R, Van NT, Mulcahy RT, et al (1998) Expression of multidrug resistance protein/GS-X pump and gamma-glutamylcysteine synthetase genes is regulated by oxidative stress. J Biol Chem 273: 31075–31085. 9813007 10.1074/jbc.273.47.31075

[18] Kauffmann HM, Pfannschmidt S, Zoller H, Benz A, Vorderstemann B, et al (2002) Influence of redox-active compounds and PXR-activators on human MRP1 and MRP2 gene expression. Toxicology 171: 137–146. 11836020 10.1016/s0300-483x(01)00570-4

[19] Kurz EU, Cole SP, Deeley RG (2001) Identification of DNA-protein interactions in the 5′ flanking and 5′ untranslated regions of the human multidrug resistance protein (MRP1) gene: evaluation of a putative antioxidant response element/AP-1 binding site. Biochem Biophys Res Commun 285: 981–990. 11467849 10.1006/bbrc.2001.5262

[20] Hayashi A, Suzuki H, Itoh K, Yamamoto M, Sugiyama Y (2003) Transcription factor Nrf2 is required for the constitutive and inducible expression of multidrug resistance-associated protein 1 in mouse embryo fibroblasts. Biochem Biophys Res Commun 310: 824–829. 14550278 10.1016/j.bbrc.2003.09.086

[21] Favreau LV, Pickett CB (1991) Transcriptional regulation of the rat NAD(P)H:quinone reductase gene. Identification of regulatory elements controlling basal level expression and inducible expression by planar aromatic compounds and phenolic antioxidants. J Biol Chem 266: 4556–4561. 1900296

[22] Moinova HR, Mulcahy RT (1998) An electrophile responsive element (EpRE) regulates beta-naphthoflavone induction of the human gamma-glutamylcysteine synthetase regulatory subunit gene. Constitutive expression is mediated by an adjacent AP-1 site. J Biol Chem 273: 14683–14689. 9614065 10.1074/jbc.273.24.14683

[23] Alam J, Wicks C, Stewart D, Gong P, Touchard C, et al (2000) Mechanism of heme oxygenase-1 gene activation by cadmium in MCF-7 mammary epithelial cells. Role of p38 kinase and Nrf2 transcription factor. J Biol Chem 275: 27694–27702. 10874044 10.1074/jbc.M004729200

[24] Furukawa M, Xiong Y (2005) BTB protein Keap1 targets antioxidant transcription factor Nrf2 for ubiquitination by the Cullin 3-Roc1 ligase. Mol Cell Biol 25: 162–171. 15601839 10.1128/MCB.25.1.162-171.2005 PMC538799

[25] Zhang DD (2006) Mechanistic studies of the Nrf2-Keap1 signaling pathway. Drug Metab Rev 38: 769–789. 17145701 10.1080/03602530600971974

[26] Osburn WO, Kensler TW (2008) Nrf2 signaling: an adaptive response pathway for protection against environmental toxic insults. Mutat Res 659: 31–39. 18164232 10.1016/j.mrrev.2007.11.006 PMC2585047

[27] Lau A, Villeneuve NF, Sun Z, Wong PK, Zhang DD (2008) Dual roles of Nrf2 in cancer. Pharmacol Res 58: 262–270. 18838122 10.1016/j.phrs.2008.09.003 PMC2652397

[28] Hayes JD, McMahon M (2009) NRF2 and KEAP1 mutations: permanent activation of an adaptive response in cancer. Trends Biochem Sci 34: 176–188. 19321346 10.1016/j.tibs.2008.12.008

[29] Hayes JD, McMahon M (2006) The double-edged sword of Nrf2: subversion of redox homeostasis during the evolution of cancer. Mol Cell 21: 732–734. 16543142 10.1016/j.molcel.2006.03.004

[30] Kensler TW, Wakabayashi N (2010) Nrf2: friend or foe for chemoprevention? Carcinogenesis 31: 90–99. 19793802 10.1093/carcin/bgp231 PMC2802668

[31] Wang XJ, Sun Z, Villeneuve NF, Zhang S, Zhao F, et al (2008) Nrf2 enhances resistance of cancer cells to chemotherapeutic drugs, the dark side of Nrf2. Carcinogenesis 29: 1235–1243. 18413364 10.1093/carcin/bgn095 PMC3312612

[32] Zhang P, Singh A, Yegnasubramanian S, Esopi D, Kombairaju P, et al (2010) Loss of Kelch-like ECH-associated protein 1 function in prostate cancer cells causes chemoresistance and radioresistance and promotes tumor growth. Mol Cancer Ther 9: 336–346. 20124447 10.1158/1535-7163.MCT-09-0589 PMC2821808

[33] Homma S, Ishii Y, Morishima Y, Yamadori T, Matsuno Y, et al (2009) Nrf2 enhances cell proliferation and resistance to anticancer drugs in human lung cancer. Clin Cancer Res 15: 3423–3432. 19417020 10.1158/1078-0432.CCR-08-2822

[34] Hong YB, Kang HJ, Kwon SY, Kim HJ, Kwon KY, et al (2010) Nuclear factor (erythroid-derived 2)-like 2 regulates drug resistance in pancreatic cancer cells. Pancreas 39: 463–472. 20118824 10.1097/MPA.0b013e3181c31314 PMC3506252

[35] Shim GS, Manandhar S, Shin DH, Kim TH, Kwak MK (2009) Acquisition of doxorubicin resistance in ovarian carcinoma cells accompanies activation of the NRF2 pathway. Free Radic Biol Med 47: 1619–1631. 19751820 10.1016/j.freeradbiomed.2009.09.006

[36] Kim SK, Yang JW, Kim MR, Roh SH, Kim HG, et al (2008) Increased expression of Nrf2/ARE-dependent anti-oxidant proteins in tamoxifen-resistant breast cancer cells. Free Radic Biol Med 45: 537–546. 18539158 10.1016/j.freeradbiomed.2008.05.011

[37] Nioi P, Hayes JD (2004) Contribution of NAD(P)H:quinone oxidoreductase 1 to protection against carcinogenesis, and regulation of its gene by the Nrf2 basic-region leucine zipper and the arylhydrocarbon receptor basic helix-loop-helix transcription factors. Mutat Res 555: 149–171. 15476858 10.1016/j.mrfmmm.2004.05.023

[38] Allen JD, Brinkhuis RF, van Deemter L, Wijnholds J, Schinkel AH (2000) Extensive contribution of the multidrug transporters P-glycoprotein and Mrp1 to basal drug resistance. Cancer Res 60: 5761–5766. 11059771

[39] Lorico A, Rappa G, Flavell RA, Sartorelli AC (1996) Double knockout of the MRP gene leads to increased drug sensitivity in vitro. Cancer Res 56: 5351–5355. 8968083

[40] Hooijberg JH, Broxterman HJ, Kool M, Assaraf YG, Peters GJ, et al (1999) Antifolate resistance mediated by the multidrug resistance proteins MRP1 and MRP2. Cancer Res 59: 2532–2535. 10363967

[41] van Tellingen O, Buckle T, Jonker JW, van der Valk MA, Beijnen JH (2003) P-glycoprotein and Mrp1 collectively protect the bone marrow from vincristine-induced toxicity in vivo. Br J Cancer 89: 1776–1782. 14583783 10.1038/sj.bjc.6601363 PMC2394394

[42] Wijnholds J, Evers R, van Leusden MR, Mol CA, Zaman GJ, et al (1997) Increased sensitivity to anticancer drugs and decreased inflammatory response in mice lacking the multidrug resistance-associated protein. Nat Med 3: 1275–1279. 9359705 10.1038/nm1197-1275

[43] Meschini S, Calcabrini A, Monti E, Del Bufalo D, Stringaro A, et al (2000) Intracellular P-glycoprotein expression is associated with the intrinsic multidrug resistance phenotype in human colon adenocarcinoma cells. Int J Cancer 87: 615–628. 10925353

[44] Shapiro AB, Fox K, Lee P, Yang YD, Ling V (1998) Functional intracellular P-glycoprotein. Int J Cancer 76: 857–864. 9626353 10.1002/(sici)1097-0215(19980610)76:6<857::aid-ijc15>3.0.co;2-#

[45] Jungsuwadee P, Nithipongvanitch R, Chen Y, Oberley TD, Butterfield DA, et al (2009) Mrp1 localization and function in cardiac mitochondria after doxorubicin. Mol Pharmacol 75: 1117–1126. 19233900 10.1124/mol.108.052209 PMC2672805

[46] Rajagopal A, Simon SM (2003) Subcellular localization and activity of multidrug resistance proteins. Mol Biol Cell 14: 3389–3399. 12925771 10.1091/mbc.E02-11-0704 PMC181575

[47] Dewa Y, Nishimura J, Muguruma M, Jin M, Saegusa Y, et al (2008) beta-Naphthoflavone enhances oxidative stress responses and the induction of preneoplastic lesions in a diethylnitrosamine-initiated hepatocarcinogenesis model in partially hepatectomized rats. Toxicology 244: 179–189. 18164116 10.1016/j.tox.2007.11.010

[48] Pietsch EC, Chan JY, Torti FM, Torti SV (2003) Nrf2 mediates the induction of ferritin H in response to xenobiotics and cancer chemopreventive dithiolethiones. J Biol Chem 278: 2361–2369. 12435735 10.1074/jbc.M210664200

[49] DeNicola GM, Karreth FA, Humpton TJ, Gopinathan A, Wei C, et al (2011) Oncogene-induced Nrf2 transcription promotes ROS detoxification and tumorigenesis. Nature 475: 106–109. 21734707 10.1038/nature10189 PMC3404470

